# Standardization of evaluation method and prognostic significance of histological response to preoperative chemotherapy in high-grade non-round cell soft tissue sarcomas

**DOI:** 10.1186/s12885-022-09195-y

**Published:** 2022-01-21

**Authors:** Yoshinao Oda, Kazuhiro Tanaka, Takanori Hirose, Tadashi Hasegawa, Nobuyuki Hiruta, Masanori Hisaoka, Masato Yoshimoto, Hiroshi Otsuka, Hirofumi Bekki, Takeaki Ishii, Makoto Endo, Toshiyuki Kunisada, Toru Hiruma, Hiroyuki Tsuchiya, Hirohisa Katagiri, Yoshihiro Matsumoto, Akira Kawai, Robert Nakayama, Hiroyuki Kawashima, Satoshi Takenaka, Makoto Emori, Munenori Watanuki, Yukihiro Yoshida, Takeshi Okamoto, Junki Mizusawa, Haruhiko Fukuda, Toshifumi Ozaki, Yukihide Iwamoto, Takayuki Nojima

**Affiliations:** 1 Department of Anatomic Pathology, Graduate School of Medical Sciences, Kyushu University, Fukuoka, Japan; 2 Department of Orthopaedic Surgery, Faculty of Medicine, Oita University, Oita, Japan; 3 Department of Diagnostic Pathology, Hyogo Cancer Center, Akashi, Japan; 4 Department of Surgical Pathology, School of Medicine, Sapporo Medical University, Sapporo, Japan; 5 Department of Surgical Pathology, Toho University Sakura Medical Center, Sakura, Japan; 6 Department of Pathology and Oncology, School of Medicine, University of Occupational and Environmental Health, Kitakyushu, Japan; 7 Department of Orthopaedic Surgery, Graduate School of Medical Sciences, Kyushu University, Fukuoka, Japan; 8 Department of Medical Materials for Musculoskeletal Reconstruction, Okayama University Graduate School of Medicine, Dentistry, and Pharmaceutical Sciences, Okayama, Japan; 9 Department of Musculoskeletal Tumor Surgery, Kanagawa Cancer Center, Yokohama, Japan; 10 Department of Orthopaedic Surgery, Graduate School of Medical Sciences, Kanazawa University, Kanazawa, Japan; 11 Division of Orthopaedic Oncology, Shizuoka Cancer Center Hospital, Shizuoka, Japan; 12 Department of Musculoskeletal Oncology, National Cancer Center Hospital, Tokyo, Japan; 13 Department of Orthopaedic Surgery, Keio University School of Medicine, Tokyo, Japan; 14 Division of Orthopedic Surgery, Department of Regenerative and Transplant Medicine, Niigata University Graduate School of Medical and Dental Sciences, Niigata, Japan; 15 Department of Orthopaedic Surgery, Osaka International Cancer Institute, Osaka, Japan; 16 Department of Orthopaedic Surgery, Sapporo Medical University School of Medicine, Sapporo, Japan; 17 Department of Orthopaedic Surgery, Tohoku University School of Medicine, Sendai, Japan; 18 Department of Orthopaedic Surgery, Nihon University Itabashi Hospital, Tokyo, Japan; 19 Department of Orthopaedic Surgery, Kyoto University Graduate School of Medicine, Kyoto, Japan; 20 JCOG Data Center, National Cancer Center Hospital, Tokyo, Japan; 21 Department of Orthopaedic Surgery, Okayama University Graduate School of Medicine, Dentistry, and Pharmaceutical Sciences, Okayama, Japan; 22 Department of Orthopaedic Surgery, Kyushu Rosai Hospital, Kitakyushu, Japan; 23 Department of Pathology, Graduate School of Medical Sciences, Kanazawa University, Kanazawa, Japan

**Keywords:** Histological response, Chemotherapy, Doxorubicin, Ifosfamide, Soft tissue sarcoma, Clinical trial, Prognosis, Neoadjuvant

## Abstract

**Background:**

Preoperative chemotherapy is widely applied to high-grade localized soft tissue sarcomas (STSs); however, the prognostic significance of histological response to chemotherapy remains controversial. This study aimed to standardize evaluation method of histological response to chemotherapy with high agreement score among pathologists, and to establish a cut-off value closely related to prognosis.

**Methods:**

Using data and specimens from the patients who had registered in the Japan Clinical Oncology Group study, JCOG0304, a phase II trial evaluating the efficacy of perioperative chemotherapy with doxorubicin (DOX) and ifosfamide (IFO), we evaluated histological response to preoperative chemotherapy at the central review board.

**Results:**

A total of 64 patients were eligible for this study. The percentage of viable tumor area ranged from 0.1% to 97.0%, with median value of 35.7%. Regarding concordance proportion between pathologists, the weighted kappa coefficient (*κ*) score in all patients was 0.71, indicating that the established evaluation method achieved substantial agreement score. When the cut-off value of the percentage of the residual tumor area was set as 25%, the *p*-value for the difference in overall survival showed the minimum value. Hazard ratio of the non-responder with percentage of the residual tumor < 25%, to the responder was 4.029 (95% confidence interval 0.893–18.188, *p* = 0.070).

**Conclusion:**

The standardized evaluation method of pathological response to preoperative chemotherapy showed a substantial agreement in the weighted *κ* score. The evaluation method established here was useful for estimating of the prognosis in STS patients who were administered perioperative chemotherapy with DOX and IFO.

**Trial registration:**

UMIN Clinical Trials Registry C000000096. Registered 30 August, 2005 (retrospectively registered).

## Background

Soft tissue sarcomas (STS) are a heterogenous group of rare malignant tumors with a wide spectrum in terms of histological findings, which comprise less than 1% of all malignant tumors [1]. The standard treatment of STS is based on the clinical stage of the tumor. The American Joint Committee on Cancer (AJCC)/International Union against Cancer (UICC) staging system is the most widely used for the staging [2]. Preoperative chemotherapy has been reported to be effective for localized high-risk STS [3]. The current standard preoperative chemotherapy for STS is anthracyclin-containing regimen including a combination of doxorubicin (DOX) and ifosfamide (IFO) is widely accepted [4–7].

Even when chemotherapy is effective, STS is not always reduced, as the tumor diameter sometimes gets enlarged because of the expansion in tumor mass due to intratumoral hemorrhage, necrosis, edema, fibrosis, and hyalinization caused by antitumor agents. A correlation between changes in tumor size on the radiological images and patient prognosis is controversial [8, 9]. Similarly, the prognostic significance of histological response to preoperative chemotherapy in high-grade STS has not been established yet. Some studies have reported a positive association between prognosis and histological response, whereas others have reported a negative association [10–18]. Therefore, there is a growing interest in the evaluation of histological effects in an attempt to assess prognosis after chemotherapy.

One of the problems is that a detailed standard evaluation method of histological response after chemotherapy on STS has not been established; therefore, differences in judgment among pathologists are likely to occur. There is no specific definition for determining whether tumor cells are viable or non-viable. The tumor cells show a wide variety of degenerative histological findings including pyknosis, vacuolation, hypertrophy and karyorrhexis, or eosinophilic change of cytoplasm. Evaluating viability of the degenerated tumor cells is often difficult, and thus it is important to define strict evaluation criteria for this purpose.

Another problem is that each paper has adopted different cut-off values to analyze the association of specific factors, such as necrosis or residual tumor, with prognosis. Some papers defined a cut-off value as 95% of necrotic proportion, others used that of 75% necrotic proportion or that of 50% residual tumor proportion. No standard cut-off value to assess the prognostic effect of histological response to chemotherapy has been established yet. In addition, information on preoperative treatment such as the type of drugs used or dose administered varies widely even within a single study. Furthermore, it was not clear whether combining radiotherapy or regional hyperthermia with chemotherapy were different among different studies conducted on the preoperative treatment because almost all previous studies were retrospective in nature.

Therefore, the aims of this study were to establish definite criteria for evaluating histological response to chemotherapy, which has high concordance rate among pathologists, and to determine a cut-off value for the percentage of residual viable tumor cells, which shows the smallest p value for the difference in patient survival.

## Patients and Methods

### Ethical statement.

All methods were carried out in accordance with relevant guidelines and regulations. This study (the Japan Clinical Oncology Group study, JCOG0304-A1) protocol was approved by the Protocol Review Committee of the JCOG and by the Institutional Review Boards in each of the 20 participating institutes.

### Patients

JCOG0304A1 is an accompanying research of a phase II trial evaluating the efficacy of perioperative chemotherapy with DOX and IFO for localized high-grade STSs in the extremities (JCOG0304) [6, 7]. All patient data and specimens used in this study were obtained from the patients who had registered in JCOG0304. JCOG0304 was conducted by the Bone and Soft Tissue Tumor Study Group of the JCOG. The details of eligibility criteria of JCOG0304 have been published previously [6, 7, 19]. The major inclusion criteria of the trial were as follows: (1) A histological diagnosis of non-round cell STSs including undifferentiated pleomorphic sarcoma (malignant fibrous histiocytoma), fibrosarcoma, leiomyosarcoma, synovial sarcoma, liposarcoma, pleomorphic rhabdomyosarcoma or undifferentiated sarcoma using open biopsy specimen; (2) FNCLCC histological grading system [20]: Grade 2 or 3; (3) AJCC stage (the 6th edition) [21]: Stage III (T2bN0M0); (4) resectable localized tumor in the extremities; (5) no history of treatment for non-round cell STS; (6), no history of chemotherapy or radiation therapy for any cancer; (7) age between 20 and 70 years; (8) written informed consent. The patients were treated by preoperative chemotherapy with DOX (30 mg/m^2^/day, days 1 and 2) and IFO (2 g/m^2^/day, days 1 to 5), which was repeated three times every 3 weeks. Preoperative radiotherapy was not allowed in JCOG0304. The tumor was resected within 5 weeks from the last cycle of preoperative chemotherapy. Postoperatively, two cycles of DOX and IFO were carried out at intervals of 3 weeks.

Among the patients enrolled in JCOG0304 study, those whose pathological specimens of the maximum cross-sectional slice of the tumor were available after receiving at least one cycle of preoperative chemotherapy were included in this accompanying study.

### Radiological evaluation

In JCOG0304, the radiological response to preoperative chemotherapy was evaluated using magnetic resonance imaging (MRI) [6]. The radiological response was centrally reviewed and assessed according to several kinds of criteria, including the WHO criteria [22]. Briefly, in the WHO criteria, the product of the largest perpendicular diameters on the cross section of the tumor was calculated, and the responses were judged as follows: complete response (CR), no residual tumor; partial response (PR), 50% or greater decrease; stable disease (SD), less than 50% decrease or less than 25% increase; progressive disease (PD), 25% or greater increase.

### Histological evaluation

In JCOG0304A1, we evaluated histological response by evaluating the area and cellularity of the residual viable tumor cells. STSs showed a variety of histological findings when responded to chemotherapy. They included hyalinization, fibrosis, cystic change, foreign body reaction, and an aggregation of foamy cells, in addition to necrosis. We decided to count the residual viable tumor area but not necrotic area to evaluate the histological response to chemotherapy because discriminating between viable and degenerative area was considered easier than distinguishing between necrotic and degenerative area.

Viable and non-viable tumor cells were defined as follows: tumor cells showing cellular swelling, nuclear swelling, increased eosinophilia of cytoplasm, slight vacuolation were considered viable, whereas those showing any evidence of pyknosis, karyorrhexis, karyolysis, severe vacuolation, or loss of nuclear staining were considered non-viable.

**Fig.1:**
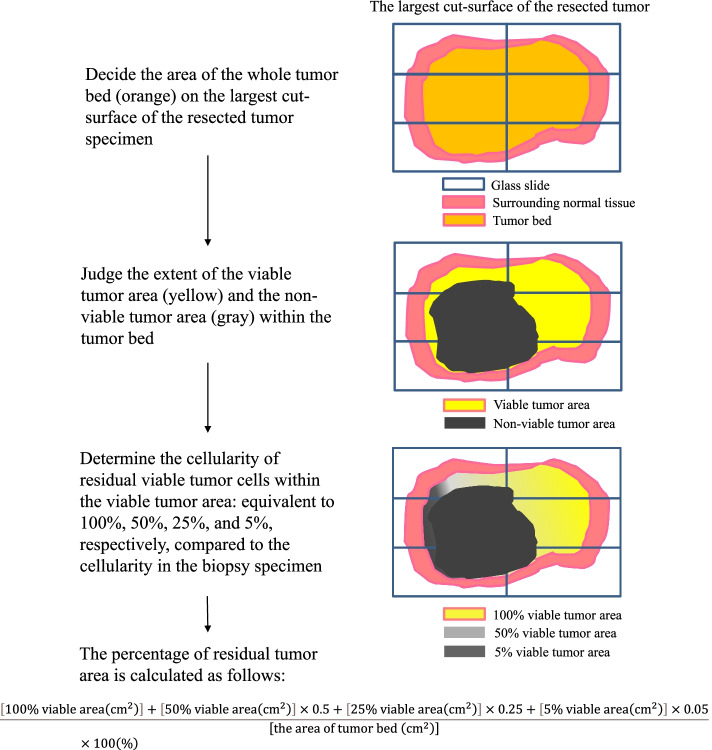
Schematic view of histological evaluation method used in this study (JCOG0304-A1)

The percentage of viable tumor area was calculated by the area in which viable tumor cells survived divided by the area of the whole tumor bed on the largest cut-surface of the resected tumor (Fig. 1). The cellularity of the residual viable cells compared to preoperative biopsy specimen was also considered. As a concrete procedure, the pathologists reviewed the virtual macroscopic slides and determined the extent of the viable area, and then marked them in the printed macroscopic picture of the cut-surface of the tumor. In addition, the tumor bed, in which the tumor was likely to have been present prior to preoperative chemotherapy was marked. If the viable cells were found reduced in cellularity due to chemotherapy, a biopsy specimen was used as a reference. The cellularity of residual viable tumor cells was classified into four categories: equivalent to 100%, 50%, 25%, and 5%, respectively, compared to the cellularity in the biopsy specimen. Finally, the percentage of residual tumor was calculated as follows:

$$\frac{\left[100\% viable tumor area({cm}^{2})\right]+\left[50\% viable area({cm}^{2})\right]\times 0.5+\left[25\% viable area({cm}^{2})\right]\times 0.25+\left[5\% viable area({cm}^{2})\right]\times 0.05}{[the area of tumor bed ({cm}^{2})]}\times 100(\%)$$

The percentage of residual tumor area was calculated as a continuous variable and used in the statistical analysis later.

### Central pathology review

First, six pathologists specialized in STS (YO, THiro, THa, NH, MH, and TN) discussed and determined the criteria for evaluation of pathological findings to assess the residual viable tumor cells after chemotherapy. Thereafter, 10 cases for test set (Group A) were randomly selected from the collected specimens, and each pathologist independently reviewed and scored the Group A cases. At the central pathology review, the pathologists discussed the histological findings causing mismatches according to the concordance rate of each case. Next, each pathologist independently reviewed 10 cases for the first validation set (Group B), which were randomly chosen from the remaining cases excluding those in Group A. When the concordance rate of Group B exceeded that of Group A and exceeded the minimum limit value of the concordance rate (weighted kappa coefficient [*κ*] > 0.6), the criteria for assessment were finalized and used to evaluate the remaining cases (Group C).

### Statistical analyses

With regard to case selection in each group, stratified random sampling was conducted according to histology (polymorphic cell sarcoma vs others) and histological grade evaluated by institutions (grade 0, 1 vs. 2, 3). As a measure of agreement, *κ* with multiple evaluaters [23, 24] in each pair of the six pathologists, i.e., 15 combinations, was calculated for the percentage of residual tumor cells.

Overall survival (OS) was defined as the time from enrollment to death from any cause and censored at the date of last contact for a surviving patient. The OS was estimated using Kaplan–Meier method. Univariable analysis was performed to investigate the impact on OS. Hazard ratios and *p* values were derived using Cox regression model. Continuous variable for group comparison was performed by Wilcoxon-rank sum test. The JCOG Data Center performed the statistical analyses using SAS version 9.4 (SAS Institute, Cary, NC).

## Results

### Patient characteristics

**Fig.2:**
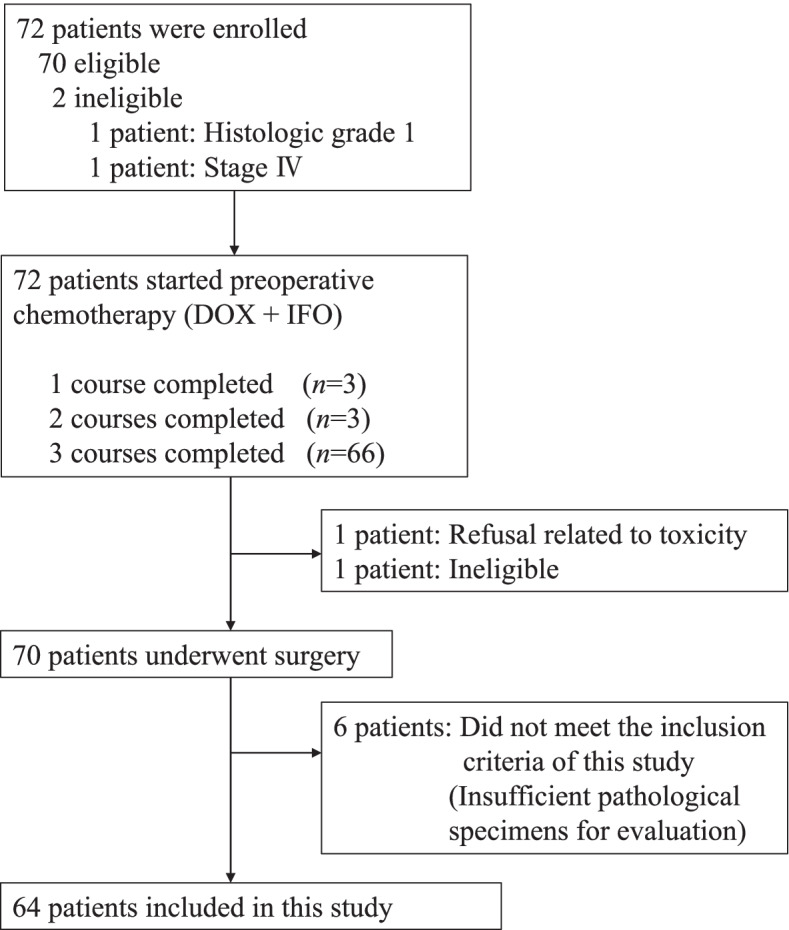
Patients flow diagram of this study (JCOG0304-A1)

From March 2004 to September 2008, 72 patients were enrolled into the JCOG0304 trial, and 64 of the 72 patients eligible for this accompanying study were included (Fig. 2). The characteristics of the included patients are listed in Table 1. Briefly, 30 patients were males and 34 were females, and the median age of patients was 47.5 years old (range 21–66). The most frequent tumor location was the thigh in 32 patients, followed by the buttock in 9 patients, the lower leg in 7 patients, the shoulder in 5 patients, the upper arm in 3 patients, the knee in 3 patients, the elbow in 2 patients, the forearm in 1 patient, the groin in 1 patient, and the ankle in 1 patient. The median tumor size was 7.4 cm. The histological diagnosis of tumors was as follows: undifferentiated pleomorphic sarcoma in 20 patients, synovial sarcoma in 17 patients, leiomyosarcoma in 12 patients, myxofibrosarcoma in 7 patients, pleomorphic liposarcoma in 3 patients, myxoid liposarcoma in 2 patients, undifferentiated/unclassified sarcoma in 2 patients, and fibrosarcoma in 1 patient.

**Table 1:** Patient characteristics (*n* = 64)

Gender	
Male/Female	30/34
Age	
Median, years	47.5
Range	21–66
ECOG	
Performance status	
0/1	45/19
Histological grade (FNCLCC)	
2/3	41/23
Site	
Shoulder	5
Upper arm	3
Elbow	2
Forearm	1
Buttock	9
Groin	1
Thigh	32
Knee	3
Lower leg	7
Ankle	1
Tumor size, major axis (cm)	
Median	7.4
Range	3.0–26.0
10 cm or less/More than 10 cm	53/11
Tumor size, minor axis (cm)	
Median	5.3
Range	1.6–13.0
Histologicacl subtype (central review)	
Undifferentiated pleomorphic sarcoma	20
Synovial sarcoma	17
Leiomyosarcoma	12
Myxofibrosarcoma	7
Pleomorphic liposarcoma	3
Myxoid liposarcoma	2
Undifferentiated/unclassified sarcoma	2
Fibrosarcoma	1
ECOG, Eastern Cooperative Oncology Group	

### Histological findings

**Fig.3:**
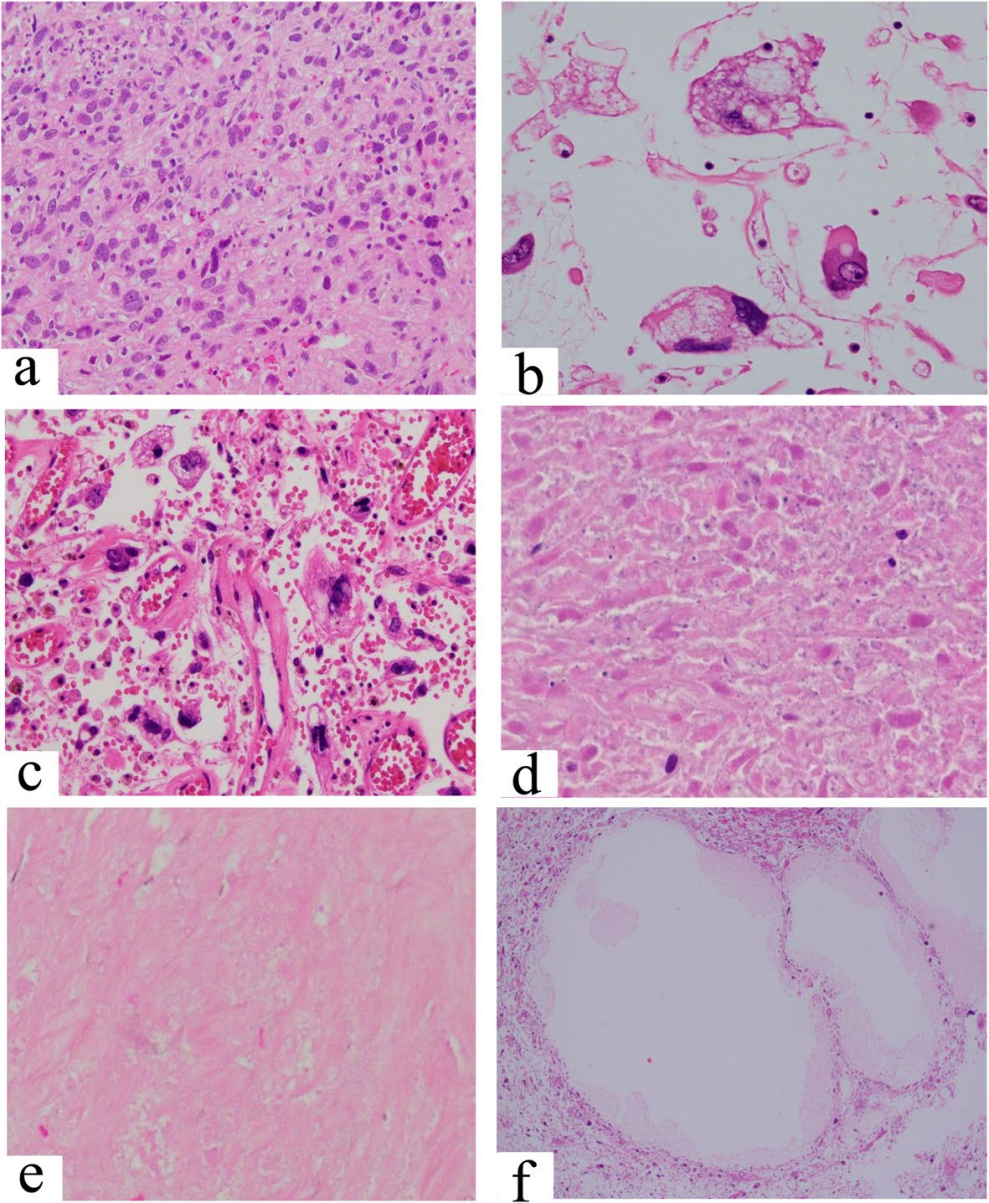
Representative histological appearance treated with preoperative chemotherapy (Hematoxylin and eosin stain, original magnification × 400): **a.** Viable tumor cells with slight nuclear swelling. **b.** Non-viable tumor cells showing degenerative change. The tumor cells changed into large, bizarre and multinucleated cells with vacuolated chromatin. **c.** Non-viable tumor cells with pyknosis, karyorrhexis and karyolysis. **d.** Necrotic tumor cells with loss of nuclear staining. **e.** fibrosis and hyalinized stroma. **f.** Intratumoral cystic change

The representative histological findings of reviewed specimens are shown in Fig. 3. The tumor specimens after chemotherapy were distinguishable into three histological areas: viable tumor area (Fig. 3a); necrotic area with irreversible nuclear changes (Fig. 3b, c, d); and degenerative area with stromal fibrosis, hyalinization, or cystic change (Fig. 3e, f). In all 64 assessed cases, the percentage of viable tumor area ranged from 0.1% to 97.0%, with median value of 35.7%.

### Concordance rate between pathologists

In 10 cases of Group A, which was the test set group, the overall weighted *κ* score was 0.73. This value met the pre-specified criteria of 0.6 and proceed to Group B. In Group B, which was the first validation set, the overall weighted *κ* score was 0.84. In the remaining 44 cases (Group C), the overall weighted *κ* score was 0.64. In total, the weighted *κ* score in all patients was 0.72.

### Association between histological response and radiological response

**Fig.4:**
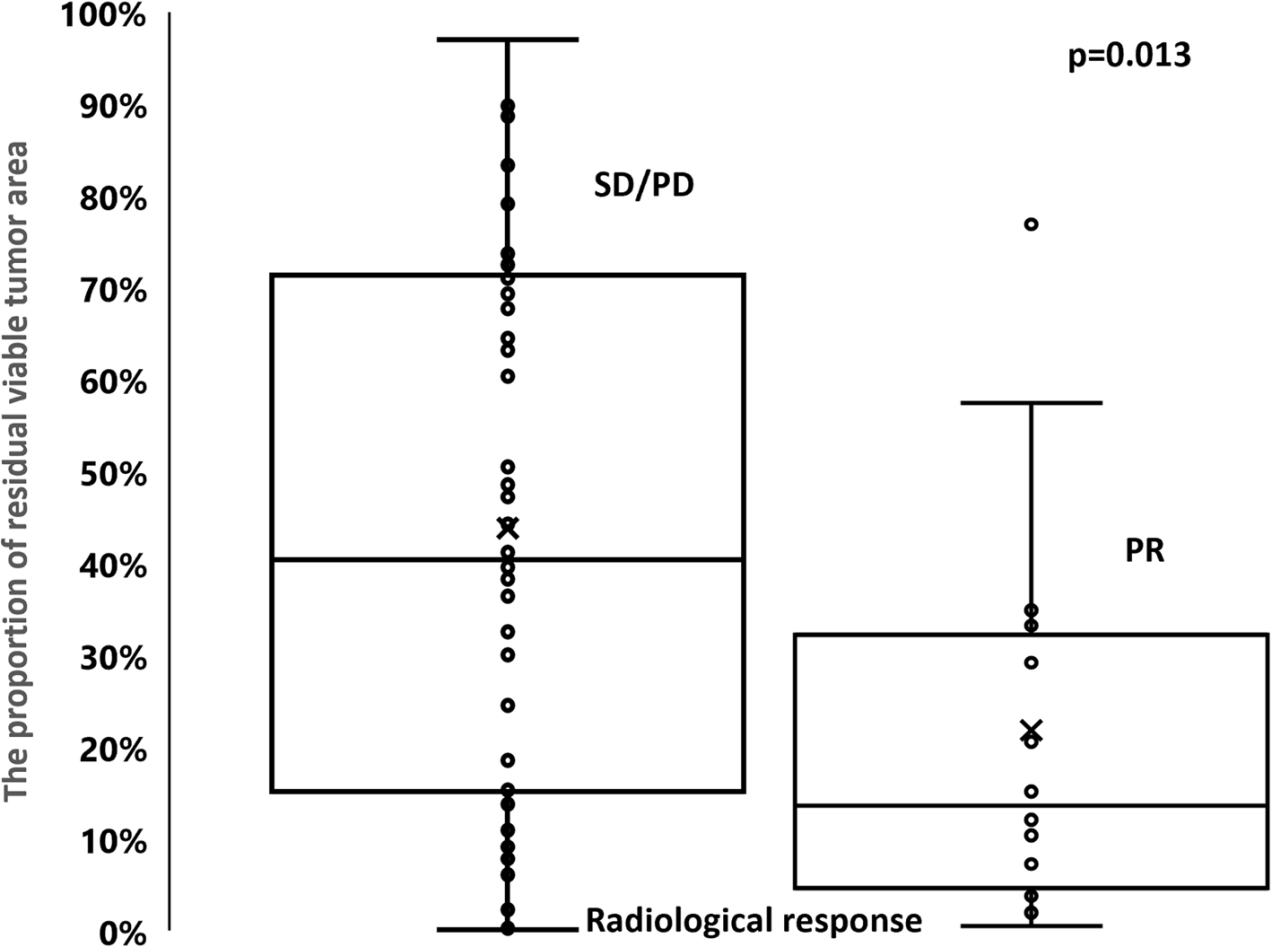
Association between histological response and radiological response. Among 64 patients included in this study, PR, SD, and PD were observed in 14, 47, and 3 patients, respectively. The median percentage of the residual tumor area was 14% in PR and 40% in non-PR (SD and PD). The percentage of the residual tumor in PR was significantly lower than that in non-PR (*p* = 0.0130). * PR: partial response; SD: stable disease; PD: progressive disease

Among 64 patients included in this accompanying study, PR in 14, SD in 47, and PD in 3 patients were observed, respectively. As shown in Fig. 4, the median percentage of the residual tumor area was 14% (interquartile range: 4%–33%) in PR and 40% (interquartile range: 15%–71%) in non-PR (SD and PD). The percentage of the residual tumor in PR was significantly lower than that in non-PR (*p* = 0.0130).

### Prognostic analysis

A median follow-up period of all cases was 10.3 years, and the shortest follow-up for survivors was 5.7 years. The 3-, 5-, and 7-year OS for 64 eligible patients was 87.5% (95% confidence interval [CI], 76.6–93.5%), 84.4% (95% CI, 72.9–91.3%) and 82.8% (95% CI, 71.0–90.1%), respectively.

**Fig.5:**
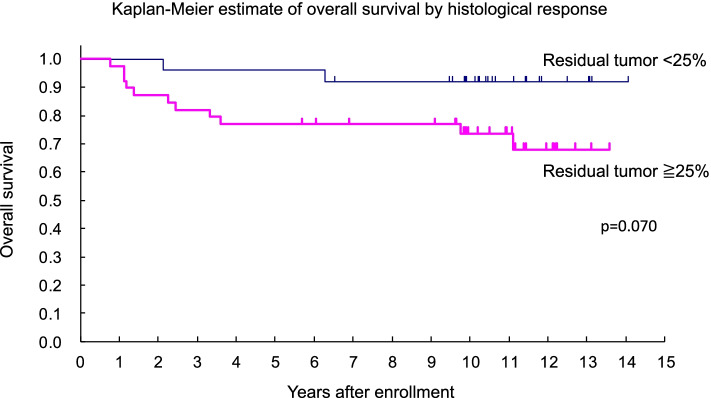
Kaplan–Meier estimate of overall survival by histological response. When the cut-off value of the percentage of the residual tumor area was set as 25%, the *p*-value for the difference in OS showed the minimum value. Hazard ratio of the non-responder (*n* = 39), whose percentage of the residual tumor was more than 25%, to the responder (*n* = 25) was 4.029 (95% CI, 0.893–18.188, *p* = 0.070)

When the cut-off value of the percentage of the residual tumor area was set between 24.565% (the largest value among the responders) and 25.150% (the smallest value among the non-responders), the *p*-value for the difference in overall survival showed the minimum value (Fig. 5). Hazard ratio of the non-responder (*n* = 39), whose percentage of the residual tumor was more than 25%, to the responder (*n* = 25) was 4.029 (95% CI, 0.893–18.188, *p* = 0.070).

## Discussion

In this study, we developed a standardized evaluation method of pathological response to preoperative chemotherapy in STS patients, which showed a substantial agreement and reproducibility score among six pathologists. The pathological response calculated here was associated with radiological response of PR or non-PR. Patients with less than 25% of the residual viable tumor area showed a trend of better OS while the prognostic results were far from conclusive.

A thorough review of the previous papers on histological response to preoperative treatment and its prognostic significance in STSs revealed non-negligible differences in regimen of preoperative chemotherapy, combination use of radiotherapy, and evaluation method of histological response (Table 2). Most of the previous studies used retrospectively collected data and small sample size. Here, we present results of a prospective analysis performed as an accompanying study of a phase II trial (JCOG0304). Compared with the previous papers, the strengths of this study (JCOG0304A1) are 1) prospective data collection, 2) long follow-up duration with a median follow-up period of over 10 years, 3) the same preoperative treatment method specified in the protocol, and 4) a central pathology review using the standardized evaluation criteria. The radiological, histological, and prognostic data used in this study were of a sufficiently high quality to deliver a critical analysis, although sample size was not large enough to conclude the prognostic significance.

**Table 2:** Comparison of JCOG0304-A1 with the previous papers

Author	Year	*n*	Preoperative chemotherapy	Concurrent preoperative therapy	Assessment	Category	Definition	5Y-OS rate	(95% CI)	10Y-OS rate	(95% CI)	*p* value	RR OS	(95% CI)	*p* value
Pezzi CM, et al	1990	27	DOX + CPA + DTIC + VCR	Radiotherapy	Necrosis, hyalinization, fibrosis, etc	Responder	Pathologic response > 0%	65%	^b^						
												*p* = 0.04			
						Non-responder	Pathologic response 0%	32%	^b^						
Issels RD, et al	2001	43	ETP + IFO + DOX	Regional hyperthermia	Necrosis	Responder	CR + PR + MR and/or necrosis ≧75%^a^	54%							
												*p* < 0.01			
						Non-responder	Other than those above	30%							
Eilber FC, et al	2001	496	DOX or DOX + CDDP or DOX + CDDP + IFO	Radiotherapy	Necrosis	Responder	Necrosis ≧95%	80%		71%			1		
												*p* = 0.0001			*p* = 0.012
						Non-responder	Necrosis < 95%	62%		55%			1.44		
Menendez LR, et al	2007	82	DOX + IFO + CDDP	None	Necrosis	Responder	Necrosis ≧95%	82%							
												NS			
						Non-responder	Necrosis < 95%	78%							
Ryan CW, et al	2008	25	EPI + IFO	Radiotherapy	Necrosis	Responder	Necrosis ≧95%	not shown							
												NS			
						Non-responder	Necrosis < 95%	not shown							
Lucas DR, et al	2008	31	DOX + IFO	No information	Necrosis, degeneration, residual tumor	Responder	Residula tumor < 50%	42%	^b^	42%	^b^				
												NS			
						Non-responder	Residual tumor ≧50%	46%	^b^	46%	^b^				
MacDermed DM, et al	2010	34	DOX + IFO or IFO or DOX + CDDP or EPI + IFO	Radiotherapy	Necrosis	Responder	Necrosis ≧90%	67.3%							
												*p* = 0.09			
						Non-responder	Necrosis < 90%	26.9%							
Donahue TR, et al	2010	55	DOX or IFO or DTIC or GEM + DTX	Radiotherapy	Necrosis	Responder	Necrosis ≧95%	83%	^c^				1	^c^	
												*p* = 0.002			*p* = 0.007
						Non-responder	Necrosis < 95%	34%	^c^				2.36	^c^	
Current study	2020	64	DOX + IFO	None	Residual tumor	Responder	Residula tumor < 25%	96.0%	(74.8–99.4)	92.0%	(71.6–97.9)		1		
(JCOG0304-A1)												*p* = 0.0498			*p* = 0.070
						Non-responder	Residual tumor ≧25%	76.9%	(60.3–87.3)	73.7%	(56.5–85.0)		4.029	(0.893–18.188)	

*DOX* doxorubicin, *CPA* cyclophosphamide, *DTIC* dacarbazine, *VCR* vincristine, *ETP* etoposide, *IFO* ifosfamide, *CDDP* cisplatin, *EPI* epirubicin, *GEM* gemcitabine, *DTX* docetaxel, *OS* overall survival, *95% CI* 95% confidence interval, *RR* relative risk, *NS* not significant

^a^Radiologic CR + PR + MR and/or pathologic necrosis ≧75%

^b^Estimated from the survial curves presented in the paper

^c^Disease-specific survival rate

Standardization of evaluation method to assess histological response to chemotherapy in STSs was one of the purposes of this study. As many as six experienced pathologists met together at a central pathology review repeatedly to establish reliable evaluation criteria. We decided to count the residual viable tumor area but not necrotic area to assess the histological response to chemotherapy because discriminating between viable and degenerative area was considered easier than distinguishing between necrotic and degenerative area. As a result, we could design the evaluation method that showed substantial concordance rate among pathologists. As for osteosarcoma, histological response to preoperative chemotherapy has been used more widely for risk stratification than STSs [25]. The most popular cut-off value between good and poor responses in osteosarcoma is 90% in necrosis, although some papers suggested the need for re-evaluation [26]. The evaluation method established in this study will be used as one of the standard procedures for the assessment of histological response to preoperative chemotherapy in STSs, and could be applied to osteosarcoma in the future research.

Our analysis indicated that the cut-off value of 25% of residual viable tumor cells showed the closest correlation with the prognosis, although the association was not statistically significant (*p* = 0.070). Our results were closest to those of Issels’s report in which the cut-off value of necrosis was set to 75% [13]. The cut-off value varied greatly among the previous papers listed in Table 2. As stated earlier, the process for setting the cut-off value has not been clearly established in previous studies. We believe that the differences in regimen of preoperative chemotherapy, concurrent therapy, included histological diagnosis, and histological evaluation method may be accountable for the differences in the cut-off value. Especially in STS, the radiological response to preoperative chemotherapy is not reliable prognostic predictor. In JCOG0304, the radiological response demonstrated no association with survival of patients with operable soft tissue sarcoma [8]. It is particularly worth noting that histological responders in our study showed an excellent prognosis, achieving up to 92.0% of 10-year OS. Prognostic prediction with our histological evaluation method could be a useful tool in the clinical management of STS patients who received preoperative chemotherapy with DOX and IFO.

There are some limitations to this study, which include technical difficulties in assessing viability of the tumor cells, presence of intratumoral necrosis prior to chemotherapy, heterogeneity of histology, and sample size. Moreover, technically, there are marginal histological differences in appearances of viable and non-viable tumor cells making it difficult to distinguish between the two. Therefore, decisions on evaluation of the tumor cell viability after severe degenerative damage caused by chemotherapeutic agents are sometimes split among pathologists. In our experience, a central pathology review helped pathologists to lower the discrepancy and to share common understandings of the pathological features. In the future, by means of distribution of plenty of histological pictures of JCOG0304A1 cases, the evaluation criteria established here should be disseminated to the pathologists who engage in the diagnosis of STS. STSs occasionally have intratumoral necrosis and hemorrhage in nature. It is therefore difficult to differentiate between necrosis caused by chemotherapy and the pre-existing one. STSs comprise of a variety of histological entities. Histology may affect the pathological response analyzed in this study. A total of seven different kinds of STS were included in this study. Finally, the number of the patients included in this study (n = 64) was not high enough to perform prognostic evaluation including multivariate analysis, even though we had a long follow-up duration and a high-quality data. We believe that further investigations with more patients are necessary to draw a more accurate conclusion.

## Conclusions

In summary, the JCOG0304-A1 evaluation method of pathological response to preoperative chemotherapy in STSs showed a substantial agreement and reproducibility score. Patients with over 25% of the residual viable tumor area tended to show better OS than others in this study. The evaluation method established here was useful for estimating the prognosis of STS patients administered perioperative chemotherapy with doxorubicin and ifosfamide.

## Data Availability

The datasets generated and/or analyzed during the current study are not publicly available due to rules of the “Japanese Ethical Guidelines for Medical and Health Research Involving Human Subjects” but are available from the corresponding author on reasonable request.

## Abbreviations

JCOG: Japan Clinical Oncology Group
STS: Soft tissue sarcoma
DOX: Doxorubicin
IFO: Ifosfamide
AJCC: American Joint Committee on Cancer
UICC: International Union against Cancer
FNCLCC: Federation Nationale des Centres de Lutte le Cancer
MRI: Magnetic resonance imaging
WHO: World Health Organization
CR: Complete response
PR: Partial response
SD: Stable disease
PD: Progressive disease
OS: Overall survival
CI: Confidence interval
